# Mr. Thelwall, on the Impediments of Speech

**Published:** 1805-05-01

**Authors:** John Thelwall

**Affiliations:** Preston, Lancashire


### *To the Editors of the Medical and Phyſical Journal.*

GENTLEMEN,

As I little imagined, several years ago, when I was attending the Anatomical and Physiological Lectures of Mr. Cline and Dr. Haighton, that I was laying thereby the foundations of Elocutionary Science, or imbibing principles that might be applied to the education of the human voice; so I can readily conceive that neither you nor your readers may be predisposed to the supposition—that the subject of this communication has any thing to do with the objects to which the Medical and Physical Journal is devoted. But impediments of speech, though originating for the most part in irregular volition, may, in some degree, be regarded as a species of disease; and it cannot be denied—that they are frequently connected (sometimes as causes and sometimes as effects) with other diseased actions of the human system. Cases, indeed, there are of this description, which come within the immediate province of the surgeon. Of this kind some have been referred to me by professional gentlemen,* while in others I have been obliged to appeal to the assistance of the operator, before the object of my instructions could be secured.† In cases, especially of defective conformation, there is assuredly much that demands the attention of the medical professor; and I should hope that the time cannot be distant when it will be thought as regular, and as honourable, for him to superintend the application of those artificial organs by which the powers of distinct and articulate utterance may be substituted for the hollow and unintelligible murmurs of a half-formed mouth, as to dictate the form and use of those mechanical implements, by which the less glorious distinctions of erect attitude, and the firm tread of human proportion, are occasionally conferred upon the miserable cripple. Even in cases where the original workmanship of Nature has not been so

* I acknowledge with respect the honour done me in this particular, by an ingenious physician, whose name will be found in another part of this paper; and to whom I am indebted for an acquaintance with several cases which throw much light on an essential part of my theory.

† Of this description were the cases of two of those young gentlemen, whose public recitations in the Town Hall of Doncaster, have given recent demonstration of the practical utilities of my science.

so deficient, circumstances have occurred that shew how intimately connected some parts of my present pursuit are with the objects of medical science; and, within these very few weeks, during my temporary residence in the neighbourhood of Doncaster, Mr. Hill, of the Dissenting College, at Rotherham, has found himself essentially relieved from some of the inconveniences of asthma by the system of management of the breath and voice, which I dictated for his improvement in the art of reading: a circumstance, indeed, which I was prepared to expect from similar advantages I had myself derived from the judicious attention paid to me in my early years, by the tutor to whom I am indebted for the first practical rudiments of my art.

These considerations embolden me to trouble you with a formal announcement of my intention to establish, in the neighbourhood of London, a College for the Cure of all Impediments of Speech, not connected with deficiency of hearing,* whether originating in mal-conformation, in accidental injuries, in mental agitation, or imitative habit; and, also, to request your insertion of the following sketch of the physiological parts of a Course of Lectures, which it is my intention at the same time to commence in the metropolis.

As the foundations of elocutionary science are equally laid in the physiological necessities that dictate the actions of the organs of speech, and in the laws of musical inflection and proportion, with which those actions most readily conform; I find it necessary to commence my course of instruction with an inquiry into the structure and offices of

 those

* With cases of deafness I do not interfere; my chief dependance being upon the ear of my pupils. But I have seen instances, and collected facts enough to induce me to warn all parents not hastily to consign their children to Colleges of the Deaf and Dumb. *Temporary* deafness, and other circumstances, during the first four or five years of infancy, sometimes produce habits, which a confusion of mind and indolent despair on the part of those who have the superintendance of such children, afterwards confirm when the cause, in reality, has ceased; and if instead of applying proper provocatives to the senses for the purpose of superinducing enunciative action, the poor child is then consigned to those silent receptacles, where inertion is confirmed by the non-presentation of objects to the proper senses, the catastrophe obviously is inevitable. What could have been more injudicious than placing the poor savage of Aveyron in the College of the Deaf and Dumb? Yet, at this very instant, a fine child, physiognomically speaking, of great promise and expectation, in the City of Carlisle, rushes upon my mind, with all the boding apprehension of a similar imolation.

those organs. These I find it necessary to distribute into two distinct classes; the *vocal organs*, which are employed in the production and variation of tunable sounds, and the *enunciative*, which are employed in superadding to those sounds the characteristic discriminations of literal and verbal expression; a classification, I believe, not hitherto observed, either by elocutionists or physiologists; and which, like other classifications, may be thought to have its difficulties; since some of the organs will, perhaps, be found to act in a double capacity of modifying the *tune*, or at least the *tone* of the voice, and of ministering to literal and verbal conformation. The distinction, nevertheless, is sufficiently obvious for all the purposes of scientific and practical application; and, certainly, in the management of impediments and deficiencies of utterance, is of sufficient importance to challenge a very minute attention. I doubt, exceedingly, whether the failure of Dr. Itard, in his attempt to confer the exercise of the faculty of speech on the Savage of Aveyron, may not, in some degree, be attributed to his overlooking this essential distinction. The organs of voice seem to have been minutely examined; though of the phenomena these should exhibit, there never appears to have been any deficiency: but what attention is recorded to the excitement of the sensibilities and varieties of lingual, labial and uvulary action, upon which the formation of verbal language must depend?

To this classification, therefore, I think it necessary to pay very particular attention; and having assumed, as my simple datum, the generally received doctrine of the origin of all sounds in percussions and vibrations of the air, I proceed to a minute investigation of the anatomy of those organs which, in the human being, give the impulses, and produce the modifications of those percussions that propagate the sounds of voice. In this part of my inquiry, I have a very powerful ally in Mr. John Gough, of Kendal; whose scientific theory, as developed in several successive papers in the Manchester Memoirs, and in his correspondence with myself upon the subject (which I communicated, by his permission, to the Monthly Magazine) falls in so exactly with the views, which, without concert or knowledge of his speculations, I had previously formed, that I instantly incorporated it with my system. Emboldened by the corroboration of his experiments, which have been further confirmed by my own reiterated repetitions, and of which sensible demonstrations are usually ally exhibited as I proceed, I endeavour not only to explain the phenomena of the variety of human voices, but to point out the means by which strength, tone, and modulation of voice may be essentially improved.

Here still my subject continues to be closely connected with Physical and Medical Science; a minute comparison of the elocutionary and the vital functions of the lungs; the requisite reception and decomposition of atmospheric air in the cells of that organ; the small portion of such air necessary for the purposes of sonorous impulse; and, above all, appeals to notorious instances of persons of the weakest and most diseased conformation attaining great command and power of voice, enabling me not only to demonstrate the importance of management and judicious tuition in these respects; but, also, the reciprocal action and re-action of vocal and constitutional improvement.

Having considered the structure and offices of the enunciative organs, in the same particular way, and demonstrated the anatomy of the elementary parts of English speech, I proceed to the primary laws of physical necessity, under which the organs act. From one simple and original principle (whose existence and operation, I trust, are sufficiently demonstrated by the series of experiments regularly exhibited) I trace the fundamental and *physical* distinctions of heavy and light syllables; and from the *unavoidable* alternations of these (or of pauses for the actions by which they should be produced) I demonstrate the formation of those simple cadences of common and triple measure, out of which arise all the beauties of rhythmus, and all the facilities of fluent and harmonious utterance. From an injudicious application of undisciplined volition to this physical action, I endeavour to account for all the gradations of harsh, ungraceful and interruptive delivery; and from inconsiderate attempts to violate this primary law, all the customary *impediments of speech.*

The practical management of these (in which consists the glory of my art) is next considered. The line of separation between organic and habitual impediment is endeavoured to be accurately marked; the distinctions of physical and moral idiotism are discussed, as far as relates to their connection with my subject; precise facts are stated (such of them as attention to the feelings of individuals will permit, with all the circumstantiality of name and place) relative to extraordinary developements and calamitous extinctions of organic capabilities; and instances are adduced of children rendered speechless by mis-manage-

 ment, ment in their early education, and of mutes who have been brought to the full exercise of the powers of speech by the application of proper stimuli. Some of the instances thus adduced have fallen under my own observation; for others I am indebted to the communications of Mr. John Gough, Dr. James of Carlisle, and other professional and scientific characters.

The chirurgical operations by which malconformations are to be remedied, constitute another ramification of this essential branch of my subject; and the structure and application of artificial organs, whose efficacy, even in the deplorable cases of fissure of the palate and obliteration of the uvula, I trust, is sufficiently demonstrable.*

The remedy of habitual impediments involves a more complicated view of my general theory. Some of these, indeed, originate in mere anatomical position; and all of them in what may be called the want of a proper understanding between the voluntary and physical powers; but the mode of treatment is so completely implicated with the musical parts of my science, that to enter into it would be to trespass upon those limits which the nature of your publication necessarily dictates to this communication. Upon that part of the subject I shall endeavour to make interest with Mr. Tillock to give insertion to a short article in his Philosophical Magazine; and those who have any curiosity to see a brief sketch of my entire plan, will probably meet with something of that kind in the cotemporary number of the Monthly Magazine; or they may find a more ample account of it, in the introductory Discourse which

---

* Of the feasibleness of this, the boldest and most difficult of all the practical applications of my science, I never myself had any kind of doubt; and the practicability of such artificial supply of the most formidable deficiency of natural organization, was, accordingly, one of the axioms I ventured to advance, at the first outset of my present lectures (between three and four years ago) when much of my subject lay yet in chaos, and many of its most essential principles were but dimly descried. But the learned and ingenious Dr. Pringle of Alnwick (to whose attentions I have many obligations to acknowledge) endeavoured to convince me, that in this respect, I carried my hypothesis to a visionary extent; and that such deficiencies were perfectly irremediable. I have since learned, that in this opinion he is completely countenanced by the first medical and anatomical characters in the metropolis. I bowed, therefore, to authority, till facts could be appealed to; and omitted, for some time, this part of my animadversions. But my visit to Birmingham presented me with the opportunity desired. Against positive demonstration, authority, however high, cannot be admitted as argument. I resume my statement, therefore, not merely as theory, but as a practical and proven fact.

which is just ready for publication. In the mean time, your insertion of the above will much oblige,

*Preston, Lancashire,*

*April* 8, 1805.

Your's, respectfully,
JOHN THELWALL.

---

### *To the Editors of the Medical and Physical Journal.*

GENTLEMEN,

